# The broader phenotypic spectrum of congenital caudal abnormalities associated with mutations in the caudal type homeobox 2 gene

**DOI:** 10.1111/cge.14076

**Published:** 2021-10-28

**Authors:** Servi J. C. Stevens, Constance T. R. M. Stumpel, Karin E. M. Diderich, Marjon A. van Slegtenhorst, Mary‐Alice Abbott, Courtney Manning, Jorune Balciuniene, Louise C. Pyle, Jacqueline Leonard, Jill R. Murrell, Romy van de Putte, Iris A. L. M. van Rooij, Alexander Hoischen, Paul Lasko, Han G. Brunner

**Affiliations:** ^1^ Department of Clinical Genetics, Maastricht University Medical Centre and GROW School for Oncology and Developmental Biology Maastricht University Maastricht the Netherlands; ^2^ Department of Clinical Genetics Erasmus Medical Centre Rotterdam the Netherlands; ^3^ Department of Pediatrics University of Massachusetts Medical School‐Baystate Springfield Massachusetts USA; ^4^ Division of Human Genetics and the Roberts Individualized Medical Genetics Center Children's Hospital of Philadelphia Philadelphia PA USA; ^5^ Department for Health Evidence, Radboud Institute for Health Sciences Radboud University Medical Center Nijmegen the Netherlands; ^6^ Department of Genetics Radboud University Medical Centre Nijmegen the Netherlands; ^7^ Department of Biology McGill University Montréal Québec Canada; ^8^ Present address: Perkin Elmer Genomics Pittsburgh PA USA

**Keywords:** caudal regression syndrome, CDX2, homeobox gene, imperforate anus, persistent cloaca, sirenomelia, VACTERL

## Abstract

The caudal type homeobox 2 (*CDX2*) gene encodes a developmental regulator involved in caudal body patterning. Only three pathogenic variants in human *CDX2* have been described, in patients with persistent cloaca, sirenomelia and/or renal and anogenital malformations. We identified five patients with de novo or inherited pathogenic variants in *CDX2* with clinical phenotypes that partially overlap with previous cases, that is, imperforate anus and renal, urogenital and limb abnormalities. However, additional clinical features were seen including vertebral agenesis and we describe considerable phenotypic variability, even in unrelated patients with the same recurrent p.(Arg237His) variant. We propose *CDX2* variants as rare genetic cause for a multiple congenital anomaly syndrome that can include features of caudal regression syndrome and VACTERL. A causative role is further substantiated by the relationship between CDX2 and other proteins encoded by genes that were previously linked to caudal abnormalities in humans, for example, *TBXT* (sacral agenesis and other vertebral segmentation defects) and *CDX1* (anorectal malformations). Our findings confirm the essential role of *CDX2* in caudal morphogenesis and formation of cloacal derivatives in humans, which to date has only been well characterized in animals.

## Graphical abstract

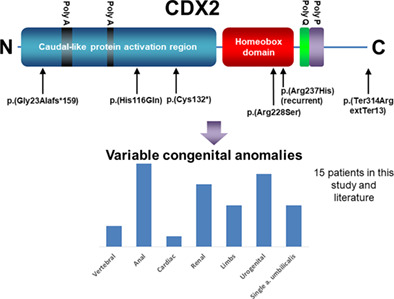

## 1 INTRODUCTION

Caudal type homeobox (*cdx*) genes encode transcriptional regulators that have a broad role in early mesodermal fate decisions and development of the body plan.^1^, ^2^, ^3^ For example, they regulate axial extension, as well as anteroposterior patterning in embryogenesis.^4^ The human genome contains three known *cdx* genes, that is, *CDX1*, *CDX2* (also known as *CDX3*), and *CDX4*, which are ParaHox genes of the HOXL subclass.^5^, ^6^ The developmental role of CDX2 has been extensively studied in animal model systems. Its role in human development and disease remains less understood, although ectopic activation of the gene is involved in the development of some cancers.^7^ Furthermore the gene is involved in human caudal morphogenesis.

Only three pathogenic germline *CDX2* variants have been described in humans.^8^, ^9^ De novo *CDX2* variants have been reported in two individuals with persistent cloaca. Inherited *CDX2* variants were identified in two families with extremely variable phenotypes that ranged from imperforate anus, renal agenesis and urogenital malformations to the most severe form of caudal abnormality sirenomelia, a malformation sequence characterized by fused legs and visceral abnormalities.^9^

Here, we describe five additional patients with pathogenic variants in the *CDX2* gene. We show that the associated phenotypic spectrum is broad and occasionally extends beyond caudal abnormalities. These findings highlight the pivotal role of the *CDX2* gene in the development of the uro‐recto‐genital tract, vertebrae, and the limbs in humans.

## 2 MATERIALS AND METHODS

Whole exome sequencing was performed as described previously^10^ using DNA isolated according to standard procedures from blood, chorion villi or skin biopsies. Exome capture was done using the Agilent SureSelect XT Human All Exon kit (Agilent, Santa Clara, CA; patient 1 and 4), the Agilent Sureselect Clinical Research Exome (CRE) Capture kit (patient 2) or the Nimblegen SeqCap_EZ_Exome_v3 kit (Roche Nimblegen, Pleasanton, CA; patient 3). Exome libraries were sequenced on an Illumina HiSeq instrument (Illumina, San Diego, CA). Sequence reads were aligned to the hg19 reference genome using BWA version 0.5.9‐r16 or Novoalign version 3. A mean coverage was obtained of 111x (patient 1), 56.5x (patient 2), 174x (patient 3) and 121x (patient 4), with at least 99.3%, 96.5%, 98.6%, and 96% of exome nucleotides covered by at least 10 sequence reads respectively. Variants were subsequently called by the GATK unified genotyper, version 3.2‐2 or higher version and annotated using custom diagnostic annotation pipelines as described previously^10^, ^11^ or by Cartagenia software (Agilent Technologies). Variants were filtered using a frequency of <1% in dbSNP and the Genome Aggregation Database (gnomAD). Data were subsequently filtered for homozygous, compound heterozygous variants or X‐linked inheritance modes, and for the de novo inheritance in parent‐offspring trio data. *CDX2* gene variants were reported by our laboratories in the routine diagnostic genetic work‐up of the patients involved in this study.

Variants in the *CDX2* gene are described for reference sequence NM_001265.5, which encodes for the CDX2 reference protein NP_001256.4, using HGVS nomenclature (www.hgvs.org). Population frequencies for variants were obtained from gnomAD (gnomad.broadinstitute.org). In silico predictions of pathogenicity for amino acid substitutions was done using Provean (provean.jcvi.org).

The patients in this study were recruited via matching submissions for the *CDX2* gene to the Genematcher website.^12^ Description of the patients' clinical phenotype was done by the consulting Clinical Geneticists as part of the routine genetic work‐up according to standard procedures for this medical profession. Parents were investigated either in a whole exome sequencing trio analysis,^10^ or via standard Sanger sequencing for the reported variant. This study was approved by the local institutes under the realm of routine diagnostic genetic testing. Patients' parents were counseled by a clinical geneticist and gave informed consent for the diagnostic procedure. Written informed consent was obtained from the patients' parents for inclusion of genotypic and phenotypic data in this study. The study conformed to principles outlined in the Helsinki Declaration.

## 3 RESULTS

Patient 1 is a 6‐year‐old girl who presented with absence of the coccygeal vertebra, anal atresia, ectopic position of a kidney and a l atrial septal defect. Whole exome sequencing of proband and parents identified a heterozygous de novo c.684G>C; p.(Arg228Ser) variant in the *CDX2* gene, which was confirmed by Sanger sequencing. The variant affects a highly conserved amino acid residue (conserved in evolution as far as *Caenorhabditis elegans*), that is located in the homeobox (HOX) domain of the CDX2 protein. A paralogous arginine residue is present in the HOX domain of most other proteins from the HOXL subclass. In CDX2, this Arg228 residue is directly involved in binding to methylated CpG islands of its target DNA.^13^, ^14^

Postpartum inspection by X‐ray of patient 2 (a foetus) showed abnormalities of the radial bones and bilateral bowed ulnae Autopsy showed bilateral cheilognathopalatoschisis, oligodactyly and abnormal position of the wrist. Whole exome sequencing of the foetus and parents identified a de novo c.348C>A; p.(His116Gln) variant in *CDX2*, which affects an evolutionarily conserved amino acid residue in the caudal‐like transcriptional activation domain of the protein. According to local policy, no Sanger confirmation was necessary as the coverage (41x) and mapping quality were sufficient for the variant.

Patient 3 is a foetus with absence or anomalies of the lower extremities, absence of one of the distal long bones, at foot and the bladder, a single umbilical artery, mild lateral curvature of the spine and a cystic mass in pelvis. Whole exome sequencing of the foetus and parents showed a de novo variant in *CDX2*, that is, c.68delG; p.(Gly23Alafs*159), which was confirmed by Sanger sequencing. This variant is located in exon one and leads to a frameshift and premature termination codon in the *CDX2* transcript. The premature termination codon is predicted to result in nonsense‐mediated decay (NMD) of the transcript, which results in haploinsufficiency, although the in vivo effect of this variant cannot be assessed with certainty. If NMD is bypassed, the premature termination codon would probably yield a non‐functional protein.

Patient 4 is a 13‐month‐old girl with a history of preterm delivery (at 30 weeks of gestation), left sided pyelectasis (resolved), umbilical cyst (resolved), and possible bladder septation/duplication. Pregnancy was complicated by maternal cystic fibrosis and well controlled Type 1 diabetes. Whole exome sequencing data for the CFTR gene did not confirm a genetic diagnosis of cystic fibrosis in the proband. Her postnatal work‐up revealed hydrometrocolpos with uterine didelphys, duplicate ovaries, septate vagina, bilateral hydroureteronephrosis and suspected clitoromegaly. Whole exome sequencing identified a variant of unknown significance in the *CHD1L* gene (NM_004284.5:c.11C>T; p.(Ala4Val) and a heterozygous c.710G>A; p.(Arg237His) variant in the *CDX2* gene, which were confirmed by Sanger sequencing. Parental testing revealed that *CHD1L* variant was inherited from the unaffected mother and it was therefore considered not to be causative for the proband's clinical phenotype,. The *CDX2* variant however was inherited from the affected father (patient 5) who presents with a solitary kidney. A younger sibling of patient 4 passed away following notice of bilateral renal agenesis, but no genetic testing was performed. The p.(Arg237)His variant found in this family is located within the HOX domain of the CDX2 protein and affects a highly evolutionarily conserved amino acid residue located between two residues that establish contact with the target DNA sequence bound by the HOX domain. Notably, this variant has a direct effect on CDX2 target gene expression in vitro.^8^

**FIGURE 1:**
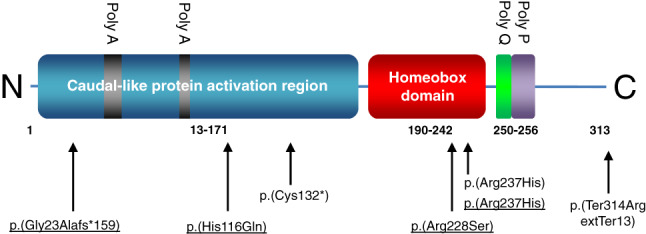
Schematic representation of the functional domains of the CDX2 proteins and the variants described in literature^8^, ^9^ and in this study (underlined). The Figure is based on CDX2 protein reference sequence NP_001256. Amino acid positions are indicated as numbers below the protein domains. The poly‐alanine (“Poly A"), poly‐glutamine (“Poly Q"), and poly‐proline (“Poly P”) stretches in the protein are indicated above the domains [Colour figure can be viewed at wileyonlinelibrary.com]

Table 1 gives details of the genotypic and phenotypic findings in the five patients compared with patients described in the literature.^8^, ^9^ Figure 1 is a schematic presentation of the *CDX2* variants described here and previously.

**TABLE 1:** Genotypic and phenotypic characteristics of patients with CDX2 variants described in this study and reported in literature^8^, ^9^

Patient	pat 1	pat 2	pat 3	pat 4	pat 5	VL6	VL21	S5‐1 (fetus)	S5‐3 (fetus)	S5‐4 (fetus)	S5‐5 (fetus)	Mother family S5	S13‐2 (fetus)	S13‐4 (fetus)	Mother family S13
Sex	Female	Male (fetus)	Female (fetus)	Female	Male	Hsu et al., Hum Mol Genet 2018		Lecoquirre et al., Hum Mutat 2020							
Age	6 years	18 weeks 6 days	12 weeks 4 days	6 day old	Father of pat 4										
Kidneys	Ectopic kidney left			History of pelviectasis bilaterally, increased echogenicity of the renal parenchyma bilaterallywithout significant cortical thinning, small lesions consistent with cysts in left kidney	Unilateral kidney				Bilateral renal agenesis		Unilateral renal agenesis			Multicystic kidneys, right kidney in pelvis	7
Upper limbs		Absent radius and hypoplastic radius, oligodactyly, bilateral absence of thumbs, abnormal position of the wrists, bowed ulnae, four metacarpalia on both sides instead of 5													
Lower limbs			Absent right lower extremity, absent left foot, absence of one of the distal long bones in the left leg							Lower limb fusion				Lower limb fusion	
Anus	Imperforate anus							Imperforate anus		Absent anus	Imperforate anus	Imperforate anus	Imperforate anus	Anus in sacral localisation	Imperforate anus
Urogenital tract			Absent bladder	Cloacal malformation with 3 cm common channel and 2 cm urethra. Duplicated ovaries, septate vagina, uterine didelphys		Persistent cloaca	Persistent cloaca		Vesical agenesis, uterus agenesis	Abnormal external genitals				Hypoplastic bladder, horizontal uterus, absent external genitals	
Umbilical cord			Single umbilical artery						Single umbilical artery	Single umbilical artery				Single umbilical artery	
Vertebrae	Absence of coccygeal vertebra		Mild lateral curvature of the spine	Normal spinal ultrasound											
Other features	Atrial septal defect	Bilateral cheilognathopalatoschisis	Cystic mass in pelvis, prenatal US also mentions “prominent bowel and prominent nuchal translucency”	Small 3rd fontanelle, overfolded helix of left ear, inverted nipples					Visceral malformations						
CDX2 variant	c.684G>C; p. (Arg228Ser)	c.348C>A; p.(His116Gln)	c.68delG; p. (Gly23Alafs*159)	c.710G>A; p. (Arg237His)	c.710G>A; p. (Arg237His)	c.396C>A; p.(Cys132*)	c.710G>A; p. (Arg237His)	c.940 T>C; p. (Ter314ArgextTer13)	c.940 T>C; p.(Ter314ArgextTer13)	c.940 T>C; p.(Ter314ArgextTer13)	c.940 T>C; p.(Ter314ArgextTer13)	c.940 T>C; p.(Ter314ArgextTer13)	c.710G>A; p.(Arg237His)	c.710G>A; p.(Arg237His)	c.710G>A; p.(Arg237His)
Inheritance mode	De novo	De novo	De novo	Familial	Familial	De novo	De novo	Familial	Familial	Familial	Familial		Familial	Familial	
CDX protein domain (missense variants)	HOX domain	Caudal ‐like protein activation domain	n.a.	HOX domain	HOX domain	n.a.	HOX domain	n.a.	n.a.	n.a.	n.a.	n.a.	HOX domain	HOX domain	HOX domain
Allele frequency of the variant in gnomAD V2.1.1 (gnomad.broadinstitute.org)	0/~246 748 alleles	0/~133 872 alleles	0/~238 834 alles	0/~203 395 alleles	0/~203 395 alleles	0/~152 962 alleles	0/~203 395 alleles	0/~247 986 alleles	0/~247 986 alleles	0/~247 986 alleles	0/~247 986 alleles	0/~247 986 alleles	0/~203 395 alleles	0/~203 395 alleles	0/~203 395 alleles
Protein Variation Effect Analyzer (Provean) in silico prediction score (provean.jcvi.org)	−5.8181 (deleterious)	−0.224 (neutral)	n.a. (frameshift variant)	−4.904 (deleterious)	−4.904 (deleterious)	n.a. (nonsense variant)	−4.904 (deleterious)	n.a. (frameshift variant)	n.a. (frameshift variant)	n.a. (frameshift variant)	n.a. (frameshift variant)	n.a. (frameshift variant)	−4.904 (deleterious)	−4.904 (deleterious)	−4.904 (deleterious)

## 4 DISCUSSION

Our findings indicate that variants in *CDX2* are a rare genetic cause for congenital abnormalities affecting the development of the anus, the renal and urogenital system, the vertebrae and/or the limbs in varying sequences and severity. We postulate that *CDX2* abnormalities cause a highly diverse and variable clinical phenotype, which shows overlap with VACTERL, that is, renal, vertebral and limb malformations and cardiac features (see Table 1). A consistent feature is uro‐recto‐genital malformation, with imperforate anus being the most frequent. The *CDX2*‐associated clinical phenotype overlaps with caudal regression syndrome, which encompasses a range of congenital defects.^15^ We propose that caudal regression syndrome, sirenomelia and persistent cloaca are part of a variable phenotypic spectrum that may also include VACTERL‐like features. A common pathogenesis for these malformations has been proposed^16^, ^17^, ^18^ and our findings may link these conditions genetically, although larger cohort studies are needed to further substantiate this.

Animal models have defined the role for CDX2 orthologues in caudal morphogenesis. The *Drosophila* caudal protein for example, is required for formation of posterior structures^19^, ^20^, ^21^ and in other arthropods the CDX2 orthologue is also required for posterior axis elongation^22^ . In *Amphioxus* the *cdx* gene is essential for gut, anus and tail patterning.^23^ In the mouse *cdx2* is essential for anteroposterior patterning of embryonal axis and morphogenesis of cloacal structures.^24^, ^25^, ^26^, ^27^ Strikingly, *Cdx2* heterozygous conditional mutant mice show a variable phenotype that can include an imperforate anus, sirenomelia, posterior vertebral truncations, and bladder anomalies,^25^, ^26^, ^28^ which is similar to the human clinical phenotype (Table 1).

CDX2 together with transcription factor T Brachyury (TBXT) co‐activates a regulatory network of target genes during posterior axial elongation and both proteins instruct the “trunk to tail” transition in mice.^29^ Strikingly, *TBXT* gene mutations in humans cause sacral agenesis and other vertebral segmentation defects,^30^, ^31^ which overlaps with the *CDX2*‐associated clinical phenotype. The clinical features also show overlap with syndromes caused by mutations in other genes of the *HOXL* subclass. For example, variants in the *MNX1* gene cause Currarino syndrome,^32^ which is characterized by sacral agenesis and imperforate anus. Variants in the *HOXL* gene *CDX1* are associated with anorectal malformations.^33^ CDX1 and CDX2 have overlapping functions in posterior axis elongation in mice^27^ and have strong co‐expression during anorectal morphogenesis in human embryos.^34^ A mutation in the *HOXL* gene *HOXD13* has been linked to VACTERL.^35^

We are unable to link the type of *CDX2* variant to the severity or diversity of the phenotype. The recurrent pathogenic missense variant in the HOX domain of the protein, p.(Arg237His), that was found in three unrelated families exhibits remarkable variability in phenotypic expression. This ranges from persistent cloaca,^8^ sirenomelia and renal/urogenital anomalies in offspring of mildly affected mothers with imperforate anus^9^ and Müllerian abnormalities in patient 4, with a solitary kidney in her mildly affected father. Patient 2 has a missense variant in the activation domain, while the other variants either concern nonsense or frameshift variants or missense variants in the HOX domain. Remarkably, patient 2 only had radial abnormalities, which are often seen in VACTERL‐like phenotypes, but caudal morphogenesis defects were absent. It remains unclear however whether this is due to location of the *CDX2* variant because the number of CDX2 patients currently is too small for a thorough genotype–phenotype analysis. Another limitation of our study is the fact that we did not perform functional or animal studies that may further define the pathogenic mechanisms causing the phenotype and may explain its variability.

The reason for the observed phenotypic diversity therefore remains unclear but may be related to (epi)genetic modifiers of the phenotype or, teratogenic environmental or maternal factors, as postulated.^18^, ^36^, ^37^ Differences in control of homeostasis of retinoic acid (RA) may possibly be involved as well. CDX2 indirectly inhibits RA by upregulating CYP26A1, a cytochrome that catabolizes RA. Loss of CDX2 function therefore leads to prolonged RA bioactivity which impairs axial mesoderm ontogenesis.^25^ Interestingly, RA exposure of heterozygous conditional mutant mice resulted in the development of sirenomelia, underscoring the molecular interplay between CDX2 and RA signaling.^25^

In conclusion, our findings confirm that *CDX2* gene variants should be considered as a rare cause of vertebral, urogenital, limb, and/or anal anomalies.

## CONFLICT OF INTEREST

The authors declare that they have no conflict of interest.

### PEER REVIEW

The peer review history for this article is available at https://publons.com/publon/10.1111/cge.14076.

## Data Availability

The data that support the findings of this study are available from the corresponding author upon request.
